# Muscle Dysfunction in Critical Illness: Established Mechanisms and the Potential Contribution of the NLRP3 Inflammasome

**DOI:** 10.3390/ijms27146114

**Published:** 2026-07-08

**Authors:** Óscar Arellano-Pérez, Joceline Arias-Díaz, Enzo Jiménez-Oliva, Denisse Valladares-Ide, Lilian Jara, Paola Llanos

**Affiliations:** 1 PhD Program in Biomedical Sciences, Institute of Biomedical Sciences, Faculty of Medicine, University of Chile, Santiago 8380453, Chile; oscar.arellano@ug.uchile.cl; 2 Public Assistance Emergency Hospital, Santiago 8320000, Chile; 3 Adult Patients Critical Centre, INDISA Clinic, Santiago 7510506, Chile; 4 Institute for Research in Dental Sciences, Faculty of Dentistry, University of Chile, Santiago 8380544, Chile; 5 Institute of Health Sciences, University of O’Higgins, Rancagua 2820000, Chile; denisse.valladares@uoh.cl; 6 Human Genetics Program, Institute of Biomedical Sciences, Faculty of Medicine, University of Chile, Santiago 8380453, Chile; ljara@med.uchile.cl

**Keywords:** muscle dysfunction, critical illness myopathy, ICU-acquired weakness, NLRP3 inflammasome, skeletal muscle

## Abstract

ICU-acquired weakness (ICUAW) is a clinical condition characterized by muscle weakness in critically ill patients that is not directly attributable to the underlying illness. It affects approximately 40% of intensive care unit patients, primarily impairing the limbs and respiratory muscles, and can compromise motor and respiratory function even after recovery from acute illness. ICUAW exhibits heterogeneous phenotypes. In addition, diverse risk factors influence its occurrence. Although this condition is recognized, the underlying mechanisms contributing to critical illness-associated muscle dysfunction remain poorly understood and are likely interrelated. This review summarizes the current experimental evidence from translational studies involving diverse muscle biopsies under various conditions, providing insights into normal skeletal muscle physiology and its alterations in critical illness-associated muscle dysfunction. Here, we focus on muscle ultrastructure, mitochondrial function, atrophy, protein breakdown, inflammation, and key molecular pathways, with consideration of the proposed role of NLRP3 inflammasome signalling, for which direct experimental evidence in human skeletal muscle during critical illness remains limited and constitutes a priority area for future mechanistic research.

## 1. Introduction

Critical illness myopathy (CIM) is a prevalent cause of intensive care unit-acquired weakness (ICUAW) [1]. It is characterized by symmetrical muscle weakness, predominantly affecting proximal muscles, while typically sparing facial and ocular muscles [2]. In addition to limb involvement, CIM can compromise respiratory muscles, contributing to ventilator-induced diaphragmatic dysfunction (VIDD), which may overlap with CIM but is a pathophysiologically distinct entity [3]. VIDD is characterized by clinical manifestations that include difficulties weaning from mechanical ventilation, highlighting its relevance within the broader spectrum of ICU-acquired weakness [3] and overlapping molecular alterations that may converge in the pathogenesis of ICUAW [4]. ICUAW may arise from critical illness polyneuropathy (CIP), a neurogenic disorder, critical illness myopathy (CIM), primary muscle dysfunction, or a combination of both conditions [5]. Given that atrophy and impaired contractility are key factors contributing to increased mortality and physical disability [6,7], an increasing number of studies has focused on CIM and its degradation pathways in skeletal muscle [8]. The development of CIM is linked to both modifiable factors, such as hyperglycaemia, corticosteroids, prolonged sedation, mechanical ventilation, parenteral nutrition, the use of vasoactive drugs, and aminoglycoside antibiotics [9,10], as well as nonmodifiable risk factors, including sepsis, systemic inflammatory response syndrome (SIRS), multiple organ failure (MOF), hyperlactatemia, and renal replacement therapy [11,12]. CIM is linked to increased mortality, prolonged intensive care unit (ICU) stays, and long-term physical disability [11,13]. It can also progress to post intensive care syndrome (PICS), leading to persistent cognitive and physical dysfunction [14,15]. CIM pathophysiology involves muscle atrophy and impaired contractility [13], resulting from an imbalance between protein synthesis and degradation, exacerbated by the ubiquitin-proteasome system (UPS), cytosolic proteases, and disrupted autophagy. Altered neuromuscular transmission, mitochondrial dysfunction, oxidative stress, and calcium homeostasis also contribute to muscle dysfunction [5]. Despite advances in understanding CIM, its precise aetiology remains unclear, highlighting the need for further research to identify therapeutic strategies. This narrative review presents findings from translational studies involving different muscle biopsies under various conditions of CIM and VIDD in critically ill patients and discusses the NLRP3 inflammasome as a biologically plausible signalling pathway that may contribute to muscle dysfunction during critical illness and requires further translational validation.

## 2. Alterations in Muscle Ultrastructure and Sarcomeric Organisation

Muscle atrophy in critically ill patients is characterized by a reduction in the fibre cross-sectional area, a decreased myosin/actin ratio, and sarcomere disruption [16,17,18,19,20,21]. Histological alterations include lipid accumulation, fibrosis, changes in myonuclear distribution, and increased lysosomal vacuolization [22,23]. These features are described in detail in the following sections.

### 2.1. Muscle Ultrastructure

**Figure 1:**
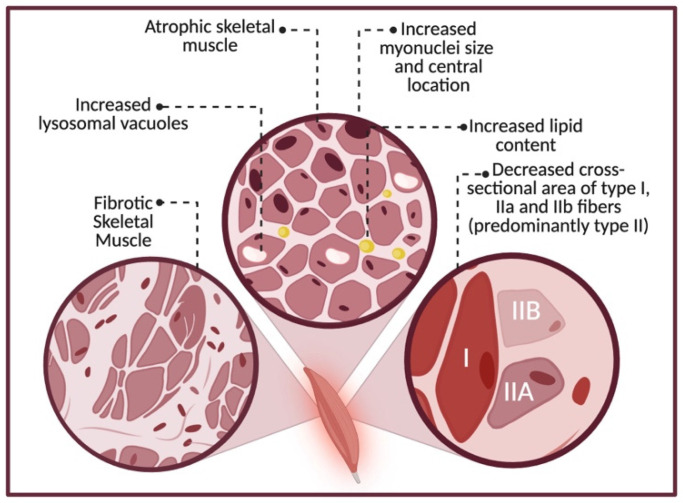
Key ultrastructural alterations reported in skeletal muscle samples from critically ill patients. These findings include muscle fibre atrophy, preferential reduction in type II fibre cross-sectional area, increased intramuscular lipid accumulation, enlarged and centrally located myonuclei, lysosomal vacuolization, and fibrotic remodelling. Collectively, these alterations are characteristic features of muscle dysfunction during critical illness. I, IIA and IIB indicate skeletal muscle fibre types I, IIA and IIB, respectively. Created in BioRender. Arellano, Ó. (2026) https://BioRender.com/0wlbb9v, accessed on 2 June 2026.

Atrophic skeletal muscle is characterized by a reduction in the cross-sectional area of fibres [24], a condition frequently observed in critically ill patients (Figure 1) [16,17,18,21,25]. This reduction is associated with the use of neuromuscular blockers and corticosteroids, as evidenced by postmortem samples from such patients [16]. However, evidence suggests that very early physical therapy can attenuate this reduction, particularly in patients with septic shock [18]. Moreover, muscle samples from critically ill patients with brain death show increased lipid content, which may reflect ongoing fibre atrophy [17]. Additionally, data from the RECOVER study cohort indicate that muscle atrophy may persist for up to six months [26].

Skeletal muscle fibres are classified into three distinct types: type I (slow-twitch and oxidative), type IIa (fast-twitch and mixed), and type IIx (fast-twitch and glycolytic) [27]. These fibre types exhibit distinct contractile properties, including contraction amplitude, tetanic strength, contraction time, and fatigability. Additionally, they differ in intrinsic characteristics, such as myosin heavy chain isoforms, ATPase activity, mitochondrial succinate dehydrogenase (SDH) activity, and sarcomeric ultrastructure [27]. Muscle atrophy in critically ill patients is fibre-type-specific. In vastus lateralis (VL) muscle samples, the cross-sectional area declines in type I, IIa, and IIx fibres, most markedly in type II fibres [19]. Similarly, tibialis anterior (TA) muscle samples from patients at increased risk for the development of ICUAW exhibit a preferential loss of type II fibres [28]. In contrast, electrical muscle stimulation (surface electrical impulses that elicit muscle contractions) has been shown to increase the cross-sectional area of fast-twitch fibres in the VL from patients with MOF and sepsis, whereas no significant changes were observed in slow-twitch fibres [29]. Paradoxically, a decrease in type I fibres has also been documented in rectus femoris biopsies from critically ill patients [30]. Fibrotic changes in skeletal muscle, marked by the presence of fibrotic fibres and extensive regeneration with basophilic fibres, were observed in TA and VL muscle samples, underscoring the profound impact of critical illness on muscle tissue [22].

Myonuclei, located beneath the sarcolemma at the periphery of muscle fibres, regulate protein synthesis within defined nuclear domains [31]. In critically ill patients, myonuclear hypertrophy with centrally located/rounded nuclei and increased lysosomal vacuoles suggests impaired autophagy [22].

### 2.2. Sarcomeric Organization

**Figure 2:**
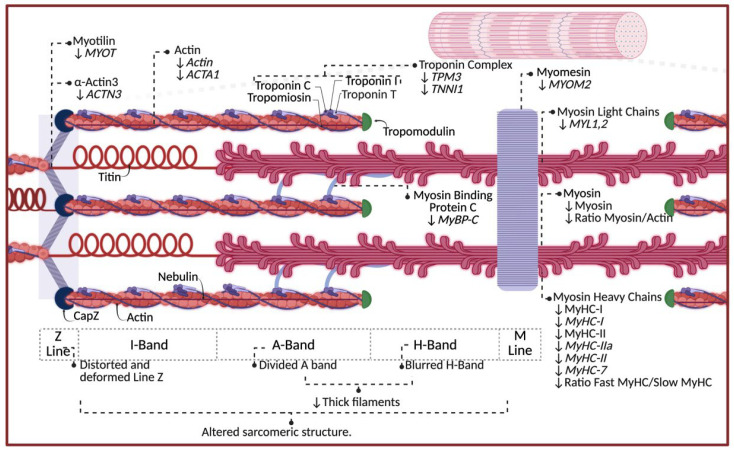
Major sarcomeric alterations reported in skeletal muscle samples from critically ill patients. The figure summarizes structural disorganization of the sarcomere, including loss of thick filaments, reduced myosin content, decreased myosin-to-actin ratio, disruption of Z-, A-, and H-band architecture, and reduced expression of several structural and contractile proteins. Collectively, these alterations are characteristic features of muscle dysfunction during critical illness and contribute to impaired force generation. Changes in mediators, indicated with nonitalic letters, refer to protein levels, whereas those in italics denote gene expression. Arrows indicate increased or decreased expression/content of the indicated proteins or genes, whereas dashed lines indicate the sarcomeric region or structural component affected. Created in BioRender. Arellano, Ó. (2026) https://BioRender.com/8z5cuaq, accessed on 2 June 2026.

The sarcomere is the fundamental contractile unit of muscle fibres and is tightly regulated, with its constituent proteins typically exhibiting well-defined localizations [32]. Several accessory proteins are redistributed within the sarcomere in response to mechanical stimuli, linking contractile activity with survival pathways, protein turnover, gene expression, and extracellular signalling [32]. In critically ill patients, the sarcomeric structure is profoundly altered (Figure 2), with loss of thick filaments, A-band fragmentation, H-band blurring and Z-line distortion [19,20,22]. In the RECOVER study, myofibrillar disarray and Z-band displacement were universal by day 7 of the ICU stay, and complete sarcomeric disruption was reported in all patients [26].

Myosin, the principal component of thick filaments in skeletal muscle, acts as the molecular motor that drives muscle contraction [32]. Its expression varies by isoform depending on the muscle fibre type [32]. Myosin depletion is a defining feature in CIM, with multiple studies reporting significant reductions in critically ill patients [2,15,33,34,35,36,37,38,39,40]. In neurocritical patients, myosin loss is particularly pronounced [41]. Additionally, decreased myosin heavy chain (MyHC) type I and IIa isoform expression is attributed primarily to corticosteroid use rather than sepsis or neuromuscular blockade [16]. Critically ill patients also exhibit a reduced myosin/actin ratio, with postmortem analyses revealing similar findings, although intensive insulin therapy appears to exert a protective effect [16]. A decline in MyHC-II and a reduced Fast MyHC/Slow MyHC ratio were linked to an increased risk of ICUAW [28]. A time-dependent decline in MyHC-I and MyHC-II isoforms has been reported, with specific decreases in MyHC1, MyHC2, and MyHC7, whereas MyHC4 levels remain unchanged [19]. Furthermore, critically ill patients also exhibit decreased expression of various MyHC isoforms alongside an increase in *MyHC-IIx/d*, likely due to passive muscle inactivity [42]. Transcriptomic analyses of skeletal muscle gene expression revealed a rapid decrease in MyHC mRNA expression and protein content, accompanied by the downregulation of myosin light chains (*MYL1*, *2*, *3*, *MYLK2*), actinin-3 (*ACTN3*), myotilin (*MYOT*), myosin-binding protein C (*MyBP-C*), and structural sarcomeric proteins such as myomesin (*MYOM*) and components of the troponin complex (*TPM3*, *TNN1*) [42]. Collectively, despite some variability among studies due to differences in the muscle groups analysed, timing of biopsy collection, and underlying clinical conditions, the available evidence consistently supports sarcomeric disorganization, myosin depletion, and a reduced myosin-to-actin ratio as hallmark features of critical illness myopathy [16,19,41]. While findings such as fibre type-specific alterations may vary across patient populations, the overall body of evidence strongly indicates that these structural abnormalities are central features of muscle dysfunction in critically ill patients.

## 3. Mitochondrial Dysfunction

**Figure 3:**
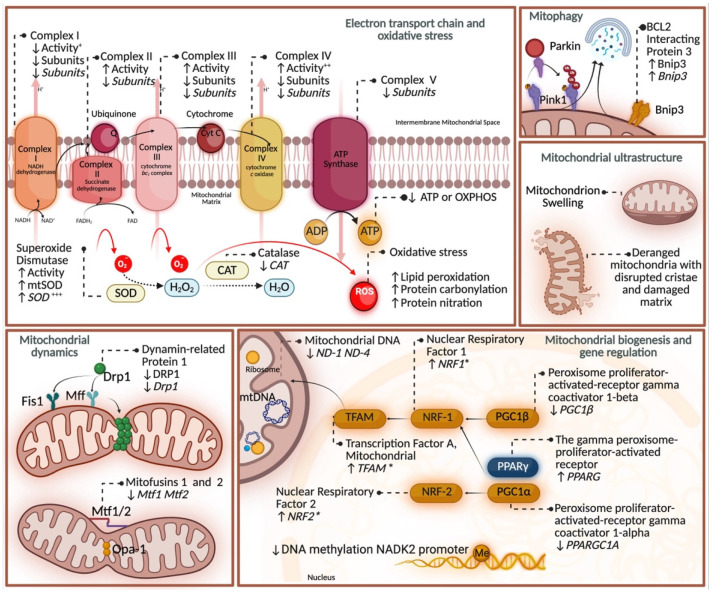
Main mitochondrial alterations reported in skeletal muscle samples from critically ill patients. These findings include abnormalities in OXPHOS complexes, reduced ATP production, increased oxidative stress, altered mitochondrial ultrastructure, dysregulated mitochondrial dynamics (fusion and fission), changes in mitophagy-related pathways, and altered regulators of mitochondrial biogenesis. Collectively, these findings indicate that alterations in mitochondrial structure, oxidative metabolism, and biogenesis are consistently observed in skeletal muscle during critical illness and are associated with muscle dysfunction, although their precise mechanistic contribution remains incompletely understood. Changes in mediators, indicated with nonitalic letters, refer to protein levels, whereas those in italics denote gene expression. Arrows indicate increased or decreased expression/content or activity of the indicated proteins, genes or mitochondrial processes; arrowed lines indicate functional relationships or signaling pathways; dashed lines indicate the mitochondrial region, complex, or structural/process component affected. ^+^, Increased activity in survivors of sepsis [20]; ^++^, decreased activity in patients with sepsis-induced multiple organ failure [44]; ^+++^, decreased expression in postmortem diaphragm muscle samples [17]; *, decreased expression in muscle samples from nonsurvivor patients with multiple organ failure (MOF) and in postmortem diaphragm muscle samples [17,20]. Created in BioRender. Arellano, Ó. (2026) https://BioRender.com/9fmgors, accessed on 2 June 2026.

Mitochondrial dysfunction plays a crucial role in the pathophysiology of CIM and VIDD, contributing to muscle damage through alterations in mitochondrial ultrastructure, dynamics, mitophagy, oxidative phosphorylation (OXPHOS) and biogenesis (Figure 3). The following sections highlight these key features in detail.

### 3.1. Mitochondrial Ultrastructure

The mitochondrial ultrastructure refers to the organization of its membranes and compartments. Mitochondria are enclosed by the outer (OMM) and inner (IMM) membranes, which are separated by the intermembrane space, which is essential for generating the proton gradient that drives ATP synthesis [43]. The IMM forms invaginations that define the inner boundary membrane, the crista junction, and the crista membrane [43]. The mitochondrial matrix, the largest aqueous compartment, contains Krebs cycle enzymes, mitochondrial DNA (mtDNA), and the transcription/translation machinery [43]. The evidence indicates that the mitochondrial ultrastructure is disrupted in CIM patients. Muscle samples from critically ill patients with multiple organ failure and sepsis exhibit altered crista morphology and varying degrees of matrix damage [44]. In VL muscle samples from patients with severe sepsis or septic shock, mitochondria are increased in size [20] or time-dependently enlarged [19]. Together, these observations indicate that mitochondrial ultrastructural disruption is a consistent morphological feature across different CIM phenotypes; however, whether these changes are causally linked to muscle dysfunction or reflect a secondary response to metabolic stress during critical illness remains unclear and represents an important gap in the current understanding of CIM pathophysiology.

### 3.2. Mitophagy

Mitophagy is a selective autophagic process that removes damaged mitochondria to preserve cellular homeostasis [45]. This process is regulated primarily by the ubiquitin/PTEN-induced kinase 1 (PINK1)/PARKIN pathway. Under normal conditions, PINK1 is degraded, but upon mitochondrial damage, it accumulates on the OMM, activating PARKIN, which ubiquitinates OMM proteins to target the mitochondrion for autophagic degradation [45]. Another OMM protein, BCL2 E1B protein-interacting protein 3 (Bnip3), acts as an autophagy receptor by directly binding to LC3, facilitating mitophagy independently of ubiquitination [45]. Elevated Bnip3 mRNA levels were detected in VL muscle samples from patients with sepsis [18,46] and in postmortem diaphragm muscle samples [4]. Additionally, an increase in the protein level of Bnip3 was observed in the VL [23], suggesting a potential association between mitophagy dysregulation and muscle pathology in critical illness.

### 3.3. Mitochondrial Dynamics

Mitochondria are highly dynamic organelles that undergo continuous fusion and fission [47]. Fission, the division of a mitochondrion into smaller units, is regulated by dynamin-related protein 1 (DRP1) [47]. Upon phosphorylation, DRP1 is recruited to the OMM, where it interacts with adaptor proteins such as mitochondrial fission factor (MFF), mitochondrial dynamics of 49 and 51 kDa (MID49/51), and mitochondrial fission 1 protein (FIS1) [47]. DRP1 assembles into a constriction ring, in coordination with dynamin 2, to mediate membrane scission and divide the organelle [47]. DRP1 expression is decreased in VL muscle samples from critically ill patients undergoing mechanical ventilation [21] and in cases of encephalic death, particularly in diaphragm muscle samples [17]. The fusion merges the OMM and IMM of two mitochondria. It is mediated by the GTPases mitofusin 1 and 2 (MFN1/2) for OMM tethering and by optic atrophy protein 1 (OPA1) for IMM fusion [47]. In diaphragm muscle samples from a critically ill patient with encephalic death, MFN1 and MFN2 transcript levels are significantly downregulated [17].

### 3.4. Mitochondrial Biogenesis and Gene Regulation

Mitochondrial biogenesis is the process by which the number of functional mitochondria increases in response to stimuli and involves the transcription of mtDNA genes and mitochondrial fission [48]. It is regulated mainly by peroxisome proliferator-activated receptor gamma coactivator 1-alpha (PGC-1α), with peroxisome proliferator-activated receptor gamma coactivator 1-beta (PGC-1β) as a secondary modulator [48]. These coactivators stimulate transcription factors such as nuclear respiratory factors 1 and 2 (NRF1/2), which increase mtDNA transcription by upregulating the expression of mitochondrial transcription factor (TFAM) [48]. Through this pathway, PGC-1α/β promote mtDNA replication, mitochondrial protein synthesis, and the import of nuclear-encoded proteins essential for OXPHOS [48]. In critically ill patients, mitochondrial biogenesis and gene regulation exhibit distinct patterns. PGC-1β transcript levels remain unchanged in VL muscle samples from patients with sepsis and MOF [49] but are reduced in diaphragm muscle samples from patients with encephalic death [17]. PGC-1α expression in VL muscle samples decreased in nonsurvivors and increased in survivors, whereas it increased in survivors of severe sepsis or septic shock [20]. A reduction in the transcript level of PGC-1α is also observed in VL muscle from patients with CIM [29] and in those undergoing mechanical ventilation [21]. NRF1 expression increases in VL muscle samples but decreases in muscle samples from nonsurviving patients with sepsis and MOF [20]. NRF1 and NRF2 are downregulated in diaphragm muscle samples from patients with encephalic death [17], whereas NRF2 is upregulated in VL from patients with sepsis [49]. Additionally, peroxisome proliferator-activated receptor gamma-2 (PPARγ2) transcript levels are elevated in the VL muscle [50]. TFAM transcript levels in the VL muscle are reduced in nonsurvivors but elevated in survivors of severe sepsis [20,49]. Conversely, TFAM transcript levels are reduced in diaphragm muscle samples from patients with encephalic death [17]. Epigenetic analyses revealed hypomethylation of CpG sites in the DNA in the *NADK2* promoter, which is associated with mitochondrial dysfunction and oxidative stress, potentially contributing to persistent muscle weakness in critically ill patients from the EPaNIC trial [50]. Furthermore, mtDNA deletions and reduced expression of mtDNA-encoded complex I subunits (ND1 and ND4) were reported in postmortem diaphragm muscle samples from patients with encephalic death [17]. These findings indicate that mitochondrial biogenesis is impaired in critically ill patients, with the downregulation of key regulatory genes leading to defective mitochondrial protein synthesis. This dysfunction may contribute to mitochondrial energy deficits, exacerbating muscle impairment, particularly in CIM and VIDD. Collectively, these findings suggest that mitochondrial biogenesis may be impaired in critically ill patients. However, the functional consequences of the observed transcriptional alterations remain incompletely understood.

### 3.5. ATP and OXPHOS

Mitochondrial ATP production, driven by the Krebs cycle and OXPHOS, is essential for cellular functions [51]. The OXPHOS system, located in the IMM, generates ATP through a series of protein complexes [51]. Complex I (CI), or NADH dehydrogenase, is the primary entry point for electron flow in the respiratory chain. It transfers electrons from NADH to ubiquinone while actively pumping protons into the intermembrane space [51]. Reduced CI activity and decreased protein levels and expression of CI subunits are reported in VL muscle samples from patients who do not survive sepsis or severe sepsis [20,52], in the external intercostal muscle from patients with sepsis and MOF [44], and in isolated mitochondria from VL muscle from patients with sepsis [49]. In contrast, CI activity is increased in VL from survivors with sepsis [20]. Complex II (CII), or succinate dehydrogenase, plays a key role in the Krebs cycle by catalysing the oxidation of succinate to fumarate, generating FADH_2_ [51]. Increased CII activity was documented in VL muscle biopsies from ventilated patients at risk of CIM [53], whereas a decrease in the transcript levels of its subunits was reported in patients with sepsis [20]. Complex III (CIII), cytochrome bc1, transfers electrons from ubiquinol to cytochrome c while contributing to proton translocation [51]. An increase in CIII activity and a decrease in protein levels of its subunits were reported in VL muscle biopsies from ventilated patients at risk of CIM [53], and decreased transcript levels of its subunits were reported in patients with sepsis [20]. Complex IV (CIV), or cytochrome c oxidase, is responsible for the final electron transfer from cytochrome c to molecular oxygen, a crucial step in aerobic respiration [51]. Increased CIV activity was observed in VL muscle biopsies from patients with sepsis [20,49], as well as in nonsurvivors [52]. Paradoxically, other studies have shown that CIV activity, as well as protein and transcript levels, is decreased in the VL of patients with sepsis, as well as in nonsurvivors [20,44,53]. Finally, complex V (CV), the mitochondrial F1F0-ATP synthase, catalyses ATP synthesis via the proton gradient [51]. The transcript levels of CV subunits are decreased in the VL of patients with sepsis [20]. Reduced OXPHOS activity has been reported in VL muscle samples from critically ill patients at risk of CIM [53], with ATP levels significantly decreased in patients with sepsis, as well as in nonsurvivors [21,44,52]. In summary, mitochondrial dysfunction, characterized by altered OXPHOS complex activity and reduced ATP production, is consistently associated with muscle wasting and poor outcomes in critically ill patients, particularly those with sepsis and at risk for CIM.

### 3.6. Oxidative Stress

Oxidative stress occurs when reactive oxygen species (ROS) production exceeds antioxidant defences [54]. Major ROS include superoxide anion (O_2_^·−^), hydrogen peroxide (H_2_O_2_), hypochlorous acid (HOCl), and nitrogen dioxide (NO_2_), which are generated during processes such as respiration and enzymatic metabolism [54]. To counteract ROS, superoxide dismutase (SOD) convert superoxide into less reactive molecules and are classified by their metal cofactors: copper-zinc (Cu/Zn-SOD), iron (Fe-SOD), manganese (Mn-SOD), and nickel (Ni-SOD) [55]. In diaphragm muscle samples from patients with encephalic death, increased lipid peroxidation and protein carbonylation are observed, indicating significant oxidative damage [4]. Mitochondrial SOD (mtSOD) activity and protein levels are elevated, but SOD activity remains unchanged in samples of the VL muscle from patients with MOF or sepsis [44,49]. Notably, MnSOD protein levels are increased in VL muscle samples from survivors of severe sepsis or septic shock but reduced in nonsurvivors [20]. Additionally, diaphragm muscle samples from patients with encephalic death exhibit upregulation of the SOD2 gene, accompanied by the downregulation of key antioxidant genes, including catalase (*CAT*), glutathione peroxidase (*GPx*), peroxiredoxin 3 (*PRDX3*), glycerol-3-phosphate dehydrogenase 1 (*GPD1*) and *SOD1* [17]. These findings suggest a dysregulated oxidative stress response, which may contribute to mitochondrial dysfunction and disease progression in critically ill patients.

Overall, the available evidence supports a central role for mitochondrial dysfunction in muscle impairment during critical illness. However, heterogeneity across studies should be acknowledged, particularly differences in the muscle groups examined, patient populations, disease severity, survival status, and timing of tissue collection. These factors may partly explain divergent findings regarding mitochondrial biogenesis markers and OXPHOS complex activity. Despite these differences, the evidence consistently points to alterations in mitochondrial ultrastructure, impaired energy production, and dysregulated oxidative stress as recurrent features of skeletal muscle dysfunction in critically ill patients.

## 4. Impaired Autophagy

**Figure 4:**
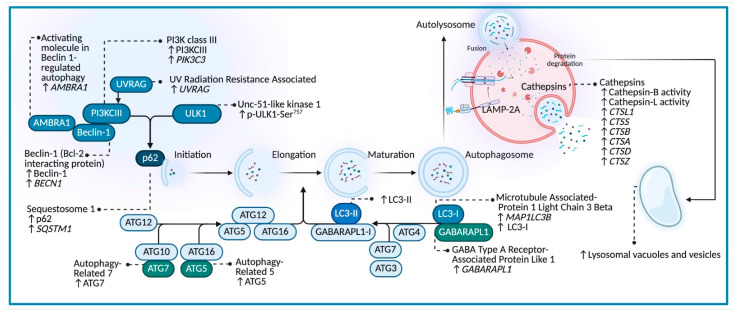
Main alterations in autophagy-related pathways reported in skeletal muscle samples from critically ill patients. Increased expression of multiple autophagy-related mediators, including Beclin-1, PI3KCIII, ATG5, ATG7, LC3, and GABARAPL1, has been described alongside accumulation of p62, lysosomal vacuoles, and altered lysosomal protease activity. Collectively, these findings suggest a complex dysregulation of autophagy characterized by activation of several autophagy-related signalling pathways despite evidence of impaired autophagic flux and lysosomal dysfunction. Changes in mediators, indicated with nonitalic letters, refer to protein levels, whereas those in italics denote gene expression. Arrows indicate increased or decreased expression/content or activity of the indicated autophagy-related mediators; arrowed lines indicate functional relationships or progression between stages of the autophagy pathway; dashed lines indicate the mediator, pathway component, or cellular process affected. Created in BioRender. Arellano, Ó. (2026) https://BioRender.com/85h5lga, accessed on 2 June 2026.

Autophagy is a key metabolic regulator in muscle that modulates energy generation, energy consumption, and macromolecule turnover [56]. However, during prolonged critical illness, insufficient autophagy impairs the clearance of damaged proteins and mitochondria [15]. This impairment is, in part, attributed to feeding-induced autophagy suppression [57]. Targeting autophagy activation through safe and specific interventions without prolonged starvation may offer novel therapeutic strategies to improve outcomes in critically ill patients [57]. The following section discusses key molecular alterations in autophagy observed in critically ill patients (Figure 4).

### 4.1. Signalling Dysfunction

The p62 which recognizes polyubiquitinated protein aggregates, facilitates their degradation by linking them to the autophagic machinery through its interaction with microtubule-associated protein 1 light chain 3 (LC3) [58]. In critically ill patients, both postmortem rectus abdominis muscle and in vivo VL muscle samples exhibit an accumulation of p62 [23]. Although increased p62 gene expression was observed in the TA muscle of patients with CIM, this increase did not reach statistical significance [42]. Transcriptional activity plays a crucial role in maintaining autophagic flux under catabolic conditions by replenishing the degradation of autophagic proteins within autophagosomes [59]. In postmortem diaphragm muscle samples from patients with encephalic death, elevated levels of autophagy-related 5 (ATG5) and autophagy-related 7 (ATG7), both of which are essential for autophagosome formation, have been reported [4]. ATG5 is critical for the elongation of the phagophore membrane, whereas ATG7 facilitates the conjugation of ATG5 to ATG12, forming a complex involved in autophagosome biogenesis [56]. Additionally, increased protein levels of the Atg5-Atg12-Bnip3 complex were detected in postmortem rectus abdominis muscle and in vivo VL muscle samples [23]. Although Bnip3 levels in the VL decrease with very early physical therapy, they remain elevated in critically ill patients compared with healthy controls, particularly in those experiencing septic shock [18]. The beclin-1 complex is regulated by various factors, including B-cell CLL/Lymphoma 2 (Bcl-2), which binds to beclin-1 and prevents its interaction with VPS34 (vacuolar protein sorting 34), a member of the PI3KCIII (class III phosphatidylinositol 3-kinase complex) family, thus inhibiting the initiation of autophagy [58]. Beclin-1 plays a crucial role in regulating the lipid kinase activity of VPS34, which generates phosphatidylinositol 3-phosphate, a key molecule required for the recruitment of autophagy proteins involved in autophagosome nucleation [58]. Thus, beclin-1 functions as an adaptor that facilitates the assembly of proteins that modulate VPS34 activity [58]. Increased protein levels of beclin-1 were reported in postmortem rectus abdominis muscle and in vivo VL muscle samples [23], and persistently high levels were detected for up to six months in the VL after critical illness [26]. Furthermore, increased beclin-1 mRNA expression was reported in postmortem diaphragm muscle samples [4]. Beclin-1 is a central component of PI3KCIII, which is essential for membrane trafficking in autophagic processes [58]. Accordingly, increased levels of PI3KCIII mRNA were identified in postmortem diaphragm muscle samples [4], alongside elevated protein levels in both postmortem rectus abdominis muscle and in vivo VL muscle samples [23]. The activating molecule in beclin-1-regulated autophagy (AMBRA1) enhances PI3KCIII activity in response to proautophagic signals by acting as a scaffold that interacts with beclin-1 [60]. Similarly, the protein product of the UV radiation resistance-associated gene (UVRAG) interacts with PI3KCIII, facilitating autophagosome maturation [56]. Consequently, increased mRNA levels of *AMBRA1* and *UVRAG* were detected in postmortem diaphragm muscle samples from critically ill patients with encephalic death [4]. Although decreased autophagic activity has been reported in CIM [23], these findings suggest the upregulation of autophagy-promoting mediators, such as beclin-1.

The unc-51-like kinase 1 (ULK1) complex initiates autophagosome formation, linking nutrient status to autophagic processes [61]. ULK1 phosphorylates several key autophagy-related targets required for autophagy initiation, including beclin-1 and VPS34 [61]. The phosphorylation status of ULK1 at specific residues can either activate or inhibit its kinase activity [61]. Compared with that in control patients, increased phosphorylation of ULK1 (Ser757) in VL muscle samples from critically ill patients with septic shock has been reported, indicating autophagy inhibition. This effect was influenced by the application of physical therapy. In contrast, p-ULK1 (Ser317), a marker of autophagy activation, was observed exclusively in the control group [18]. These findings suggest that ULK1 phosphorylation plays a critical role in regulating autophagy during critical illness and may be modulated by therapeutic interventions such as physical therapy. Nutrient-deprivation autophagy factor-1 (NAF-1) counteracts the inhibitory effect of Bcl-2 on beclin-1-dependent autophagy, playing a crucial role in the homeostatic maintenance of skeletal muscle [62]. Increased NAF-1 gene expression was observed in the TA muscle of patients with CIM [42].

LC3 is a key marker of autophagy that undergoes conjugation to phosphatidylethanolamine (PE) to form LC3-II, which is subsequently recruited to the autophagosome membrane. These autophagosomes then fuse with lysosomes to form autolysosomes, whose contents are degraded by lysosomal hydrolases [63]. Elevated LC3-II protein levels were detected in postmortem diaphragm muscle samples from patients with encephalic death [4]. Additionally, increased LC3-I protein levels were noted in both postmortem rectus abdominis muscle and in vivo VL muscle samples from critically ill patients receiving intensive insulin treatment [23]. The conversion of LC3-I to LC3-II is commonly used as a marker of autophagosome formation, but its interpretation is difficult since LC3-II is also degraded during autophagy, and LC3 levels at a given time may not accurately reflect autophagic flux, depending on the timing of measurements [64]. In contrast, LC3 mRNA levels remain elevated in the VL from critically ill patients compared with controls, particularly in those with septic shock, although they decrease with very early physical therapy [18]. Similarly, the *MAP1LC3B* gene tends to be overexpressed in the TA muscle of patients with CIM [42]. GABA type A receptor-associated protein like 1 (GABARAPL1) is an Atg8 orthologue categorized into the GABARAPL1 and LC3 subfamilies and plays a key role in autophagy by associating with autophagic vesicles [65]. GABARAPL1 expression is elevated in postmortem diaphragm muscle samples [4] and decreased in the VL of patients with sepsis undergoing very early physical therapy [18].

### 4.2. Lysosome Impairment

Lysosomes are essential components of the degradation system in mammalian cells. These membrane-bound organelles contain high concentrations of various acid hydrolases, which play crucial roles in macromolecule degradation [66]. An increase in lysosomal vacuoles and vesicles was observed in VL and TA muscle samples from patients with CIM [22]. Additionally, double-membrane vesicles, indicative of autophagosomes, were identified near the mitochondria in postmortem diaphragm muscle samples [4]. Cathepsins L, B, D, and H are key lysosomal proteases that determine the proteolytic capacity of lysosomes [66]. Elevated cathepsin L mRNA levels have been reported [4]. Moreover, increased activity of cathepsins B and L was observed in the VL of critically ill patients with sepsis [46], with significantly higher cathepsin L protein levels in patients with septic shock than in controls [18]. Additionally, upregulated expression of multiple cathepsin genes, including *CTSS*, *CTSB*, *CTSA*, *CTSD*, and *CTSZ*, was observed in the TA of critically ill patients with CIM [42]. These findings suggest that increased lysosomal protease activity, with increased protein content and gene expression levels, contributes to the dysregulation of proteolysis in critical illness. Lysosomal proteolysis involves macroautophagy, microautophagy, and chaperone-mediated autophagy (CMA), with lysosomal-associated membrane protein 2A (LAMP-2A) playing a key role in CMA-mediated substrate translocation [67]. A reduction in LAMP-2A and p62 colocalization was observed in VL muscle samples from patients with sepsis undergoing very early physical therapy, suggesting lysosomal dysfunction during critical illness [18]. In CIM, dysregulated autophagy is characterized by impaired autophagosome formation, altered protein accumulation, and increased lysosomal protease activity, all of which may contribute to excessive protein degradation [4,23,42]. However, the precise role of autophagy in this process remains controversial [57]. Despite these disruptions, the interpretation of autophagy-related alterations remains challenging. While the accumulation of p62 suggests impaired autophagic clearance, increased expression of Beclin-1, PI3KCIII, ATG proteins, and LC3-related markers may indicate activation of autophagy-related pathways. These apparently divergent findings suggest a complex and potentially dysregulated autophagic response rather than a simple increase or decrease in autophagic activity. Furthermore, differences in muscle groups analysed, clinical conditions, and timing of tissue collection may contribute to variability among studies.

## 5. Alterations in Protein Synthesis/Degradation Signalling

**Figure 5:**
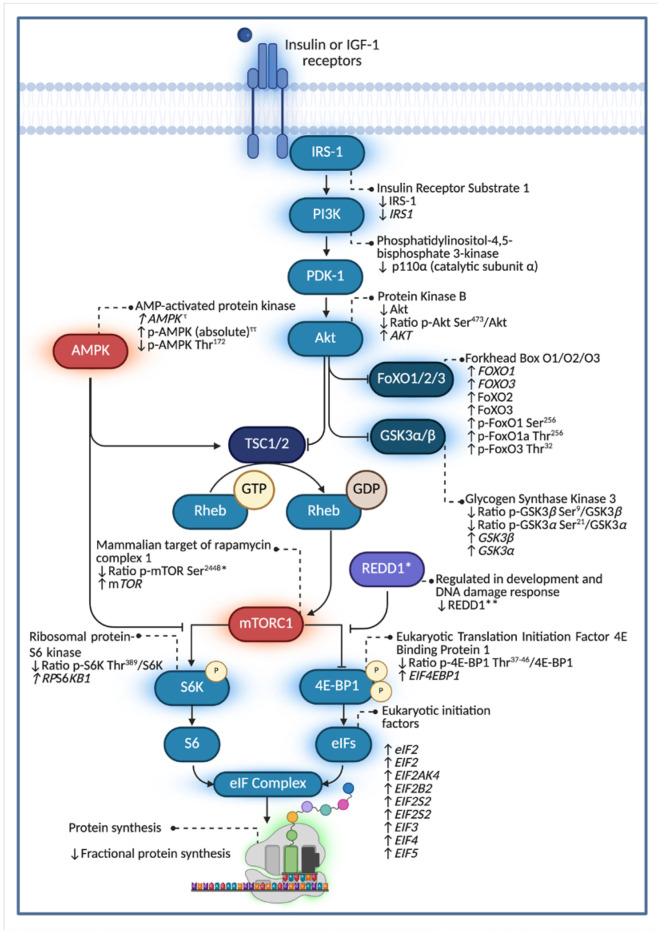
Main alterations in insulin/IGF-1/Akt/mTOR signalling reported in skeletal muscle samples from critically ill patients. The figure summarizes changes in upstream anabolic signalling (IRS-1, PI3K and Akt), regulators of protein synthesis (mTOR, S6K, 4E-BP1 and translation initiation factors), and catabolic mediators of the FoxO family. Although some signalling components exhibit heterogeneous responses across studies, the overall evidence suggests dysregulation of pathways controlling muscle protein turnover, accompanied by reduced fractional protein synthesis and activation of catabolic signalling. Changes in mediators, indicated with nonitalic letters, refer to protein levels, whereas those in italics denote gene expression. Colored circles indicate phosphorylation sites or activated signalling nodes. Arrows indicate increased or decreased gene expression, or changes in protein content, activity, or phosphorylation; arrowed lines indicate functional relationships within the insulin/IGF-1/Akt/mTOR pathway; dashed lines indicate the mediator or pathway component affected. ^τ^, decreased AMPK expression in postmortem diaphragm and VL muscle samples from critically ill patients [17,29]; ^ττ^, reduced phosphorylation in postmortem diaphragm muscle samples [17]; *, increased phosphorylation in postmortem rectus abdominis muscle samples [23]; **, and decreased protein levels with early physical therapy in patients with CIM [18]. Created in BioRender. Arellano, Ó. (2026) https://BioRender.com/d4fjkyu, accessed on 2 June 2026.

Disruptions in key components of the insulin-like growth factor 1 (IGF-1)/insulin signalling pathway contribute to muscle atrophy (Figure 5). The following subsections detail these molecular alterations and their roles in the pathogenesis of muscle dysfunction in critically ill patients.

### 5.1. Insulin-like Growth Factor 1 (IGF-1) and Insulin Pathways

The IGF-1 and insulin signalling pathways, which involve several intracellular mediators, regulate protein synthesis and muscle growth [68,69]. These pathways include insulin receptor substrates 1 and 2 (IRS-1/2), whose phosphorylation enhances metabolic responses to hormonal stimulation [68,69]. Phosphorylated IRS activates phosphatidylinositol 3-kinase (PI3K), which generates phosphoinositide 3,4,5-triphosphate (PIP3). PIP3 recruits 3′-phosphoinositide-dependent kinase 1 (PDK1), which phosphorylates Akt at Thr308, a central kinase for protein synthesis [68]. In addition, phosphorylation of Akt at Ser473 by mTORC2 (Rictor-containing mTOR complex) regulates downstream targets such as forkhead box O (FoxO) transcription factors but does not affect tuberous sclerosis complex 2 (TSC2) or ribosomal protein S6 kinase (S6K), a key regulator of protein synthesis [68]. Reduced mRNA and protein levels of IRS-1 have been reported in diaphragms from patients with encephalic death [17] and in VL muscle samples from patients with CIM [29] and MOF [49]. Furthermore, decreased protein levels of the p110α catalytic subunit of PI3K in VL muscle samples from critically ill patients with MOF and sepsis, irrespective of a CIM diagnosis, have been reported [29].

### 5.2. Akt Kinase

Akt functions as a key regulator in this cascade, promoting protein synthesis via the mammalian target of rapamycin (mTOR) and glycogen synthase kinase 3 (GSK3) while also driving muscle degradation through FoxO transcription factors [68]. In postmortem diaphragm muscle samples, a decrease in total Akt protein levels was observed [4]. Additionally, reduced p-Akt (Ser473) was reported in the VL of patients with CIM [70]. However, physiotherapy interventions in critically ill patients increase the protein levels of both Akt [29] and p-Akt (Ser473) [18]. Akt phosphorylation inhibits two isoforms of GSK3: p-GSK3β (Ser9) and p-GSK3α (Ser21). This suppression of GSK3 activity, including its inhibitory phosphorylation of eukaryotic initiation factor 2B (eIF2B), enables eIF2B to interact with the eukaryotic initiation factor 2 (eIF2) complex and facilitate translation initiation [71]. A decrease in p-GSK3α (Ser21) and p-GSK3β (Ser9), which are correlated with increased GSK3α and GSK3β mRNA levels in the VL from critically ill patients, has been reported [70].

### 5.3. Mammalian Target of Rapamycin (mTOR)

The mTOR signalling pathway is crucial for muscle maintenance, as it regulates protein synthesis, and its downregulation contributes to muscle mass loss by reducing muscle fibre size [24]. Increased mTOR mRNA levels were observed in VL from critically ill patients with sepsis [29,49]. However, discrepancies in mTOR activation have been reported. A decrease in p-mTOR (Ser2448) in VL muscle samples has been reported [70], whereas increased p-mTOR levels in postmortem rectus abdominis muscle samples have been reported, although no such increase was detected in vivo [23]. When activated, mTORC1 promotes protein synthesis by phosphorylating key effectors such as p70S6 kinase 1 (S6K1) and eIF4E-binding proteins (4E-BP). The primary target of S6K1 is ribosomal protein S6, which plays an essential role in translation initiation and muscle protein synthesis [68,69]. In VL muscle samples from critically ill patients, altered phosphorylation patterns of S6K were reported, with decreased p-S6K (Thr389) levels relative to total S6K, despite an increase in S6K mRNA levels [70]. Translation initiation is triggered by mTORC1-mediated phosphorylation of 4E-BP1, which induces the dissociation of 4E-BP1 from eIF4 at the 5′ cap of the mRNA [72]. This dissociation facilitates the interaction between eukaryotic initiation factor 4E (eIF4E) and eukaryotic initiation factor 4G (eIF4G), thereby enabling mRNA translation [72]. In VL muscle samples from critically ill patients, a decrease in p-4E-BP1 (Thr37-46) was observed, along with an increase in 4E-BP1 mRNA levels [70]. Similarly, elevated 4E-BP1 mRNA levels were reported in postmortem diaphragm muscle samples [17]. With respect to components of the eukaryotic initiation factor (eIF) complex, the upregulation of *eIF2*, *EIF2*, *EIF2AK4*, *E2B2*, and *E2S2*, as well as members of the EIF3, EIF4, and EIF5 complexes, along with ribosomal subunits in TA muscle samples from a patient with CIM, was observed [42]. Despite the presence of factors that promote protein synthesis, decreased fractional protein synthesis has been reported in the VL from CIM patients [25]. Regulated in development and DNA damage response 1 (REDD1) is a stress-induced protein that functions as an inhibitor of the mTOR pathway [73]. Early physical therapy has been shown to reduce REDD1 protein levels in the VL of patients with CIM, suggesting a potential therapeutic role in mitigating mTOR pathway inhibition [18]. Another key regulator, adenosine monophosphate-activated protein kinase (AMPK), modulates skeletal muscle growth by inhibiting mTORC1 activity [68]. However, divergent findings have been reported regarding AMPK activity in CIM. An increase in AMPK mRNA levels in VL muscle from patients with sepsis has been reported [49], whereas decreased AMPK mRNA levels in postmortem diaphragm muscle samples from critically ill patients have been reported [17]. Similarly, reduced AMPK mRNA levels in the VL from patients with sepsis and MOF have been reported [29]. With respect to AMPK protein levels, an increase in the phosphorylation of AMPK in VL muscle samples has been described [21], whereas a reduction in phosphorylation in postmortem diaphragm muscle has been observed [17]. Additionally, a decrease in p-AMPK (Thr172) in the VL muscle has been reported [18]. These contrasting findings underscore the complex and context-dependent role of AMPK in critical illness and CIM.

### 5.4. Forkhead Box O (FoxO) Family

Akt also prevents muscle degradation by phosphorylating FoxO transcription factors, which promote muscle atrophy through the upregulation of E3 ubiquitin ligases and the induction of autophagosome membrane components [74]. Increased mRNA and protein levels of p-FoxO1 (Ser256) have been reported in postmortem VL muscle samples from patients with CIM [16] and postmortem diaphragm muscle samples from brain-dead patients [4]. Additionally, time-dependent increases in FoxO1 mRNA levels were observed in VLs from critically ill patients [19], whereas other studies have demonstrated elevated protein levels of FoxO2 and FoxO3, as well as increased FoxO3 mRNA levels [16,18]. Additionally, time-dependent increases in the protein levels of p-FoxO3 (Thr32) and p-FoxO1a (Thr24) in VL from patients with CIM have been reported [18]. In summary, alterations in the insulin–IGF1/Akt/mTOR signalling pathway are consistently observed in critically ill patients and may contribute to impaired protein homeostasis and muscle atrophy. However, interpretation of these findings is complicated by substantial variability among studies. Divergent results regarding Akt, mTOR, AMPK, and downstream effectors may reflect differences in patient populations, disease severity, timing of tissue collection, muscle groups analysed, and methodological approaches. Moreover, the coexistence of increased expression of several anabolic signalling mediators with reduced fractional protein synthesis suggests that transcriptional activation does not necessarily translate into preserved protein synthesis during critical illness. Collectively, these findings support a complex dysregulation of anabolic signalling rather than a uniform suppression of protein synthesis pathways.

## 6. Ubiquitin Proteosome System

**Figure 6:**
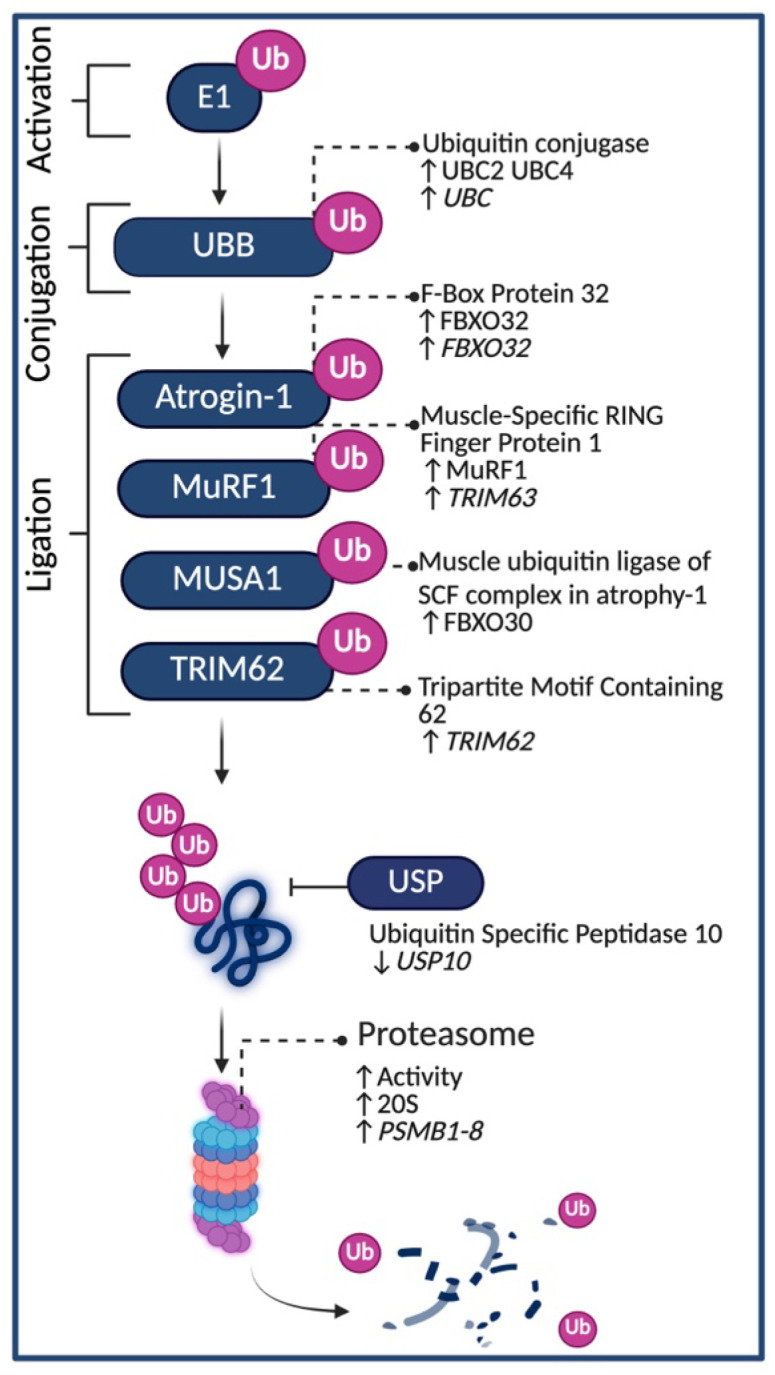
Main alterations in the ubiquitin–proteasome system (UPS) reported in skeletal muscle samples from critically ill patients. Increased expression of ubiquitin-conjugating enzymes, E3 ubiquitin ligases (including Atrogin-1, MuRF1, MUSA1, and TRIM62), and proteasome components has been consistently observed, together with reduced expression of the deubiquitinating enzyme USP10. Collectively, these findings support activation of the UPS as a prominent proteolytic pathway associated with skeletal muscle protein degradation during critical illness. Changes in mediators, indicated with nonitalic letters, refer to protein levels, whereas those in italics denote gene expression. Arrows indicate increased or decreased expression/content or activity of the indicated UPS-related mediators; arrowed lines indicate functional relationships or progression through the ubiquitin–proteasome pathway; dashed lines indicate the mediator, enzyme, or pathway component affected. Created in BioRender. Arellano, Ó. (2026) https://BioRender.com/41nmbwb, accessed on 2 June 2026.

The UPS is a crucial ATP-dependent proteolytic pathway responsible for degrading misfolded or defective proteins (Figure 6) [75]. Protein degradation is initiated by the covalent attachment of ubiquitin (Ub) molecules through an enzymatic cascade [76]. The main findings regarding the UPS in muscle samples from critically ill patients are described below.

### 6.1. Ubiquitin-Conjugating Enzymes (E2)

Initially, ubiquitin binds to the Ub-activating enzyme (E1) via a high-energy thioester bond, which is transferred to the Ub-conjugating enzyme (E2) [75]. In postmortem diaphragm muscle samples from patients with encephalic death, increased protein levels of UBC2 and UBC4 (ubiquitin-conjugating enzyme E2) were observed [4], along with elevated UBC mRNA levels in the VL from patients with sepsis [46].

### 6.2. Ubiquitin Ligases (E3s)

After E1 and E2 activity, an E3 ubiquitin ligase then catalyses the conjugation of ubiquitin to the target protein through an isopeptide bond. This process continues until a polyubiquitin chain is formed, signalling that the 26S proteasome degrades the target protein [75,77]. Muscle RING Finger 1 (MuRF1) and atrogin-1/MAFbx are two key E3 ubiquitin ligases identified as universal markers of muscle atrophy [77]. Increased mRNA levels of Atrogin-1 and MuRF1, including those in the diaphragm and quadriceps, were observed in postmortem muscle samples from critically ill patients with encephalic death [4]. These increases have also been confirmed at the protein level in VL muscle samples [70]. Studies of VL biopsies have shown early increases in Atrogin-1 [19] and MuRF1 [19,78] mRNA at the onset of critical illness, followed by a decrease over time. Additionally, upregulated mRNA levels of both Atrogin-1 and MuRF1 were associated with corticosteroid use but not with neuromuscular blockade or sepsis [16]. Increased expression of this ubiquitin ligase in the TA muscle has also been documented in transcriptomic studies [42]. Additionally, other E3 ubiquitin ligases have been implicated in the breakdown of skeletal muscle proteins, including MUSA1 (muscle ubiquitin ligase of the SCF complex in atrophy-1) [77], whose overexpression has been reported in transcriptomic analyses of muscle biopsies from critically ill patients [42]. Furthermore, elevated gene expression and protein levels of the E3 ubiquitin ligase TRIM62 (tripartite motif-containing 62) were observed in VL muscle biopsies, potentially reflecting its role in the inflammatory response in these patients [78].

### 6.3. Proteasome

The 26S proteasome, which is responsible for degrading ubiquitinated proteins [77], exhibits increased activity in CIM [46]. The proteasome consists of a cylindrical catalytic core, or the 20S proteasome, and two ATPase-containing complexes, known as 19S cap complexes, which are present in both the nucleus and the cytoplasm [75]. Elevated mRNA and protein levels of the 20S proteasome and its subunits PSMB1-8 (proteasome Subunit Beta Types 1-8) were reported in the VL of critically ill patients [70]. Additionally, deubiquitinating enzymes (USP), such as ubiquitin specific peptidase 10 (USP10), are thought to prevent UPS-mediated degradation [76]. Notably, decreased USP10 expression was reported in VL muscle samples from critically ill patients [50].

### 6.4. Caspases and Calpains: Upstream Mediators of Proteasome-Mediated Degradation

The proteasome-mediated degradation pathway specifically targets muscle proteins, including monomeric myosin, actin, and tropomyosin, but not intact myofibrils [77]. Instead, proteolytic enzymes such as calpains and caspases facilitate the initial disassembly of these structures, allowing subsequent degradation by the UPS [77]. Increased caspase-3 and m-calpain levels, along with elevated caspase-3 activity, were reported in VL biopsies from patients with septic shock [46]. Furthermore, higher caspase-3 levels were detected early in VL muscle samples from critically ill patients, with calpain-1 levels rising after 15 days in the ICU [19]. Transcriptomic analyses also revealed upregulated gene expression of various caspases and calpains in TA from patients with CIM [42].

Collectively, despite differences in patient populations, muscle groups analysed, and methodological approaches, the available evidence consistently demonstrates activation of the ubiquitin–proteasome system during critical illness. The recurrent upregulation of MuRF1, Atrogin-1, proteasome components, caspases, and calpains across independent studies suggests that enhanced proteolysis is a common molecular feature underlying skeletal muscle dysfunction in critically ill patients.

## 7. Inflammatory Responses in Critical Illness and Development of Muscle Dysfunction

**Figure 7:**
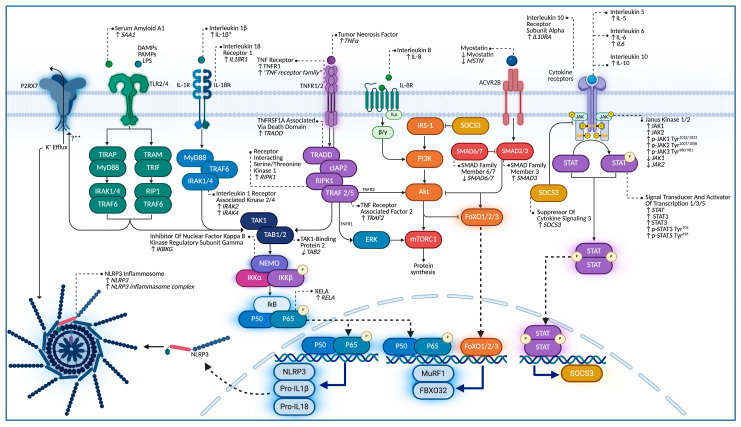
Main inflammation-related signalling pathways altered in skeletal muscle during critical illness. The figure summarizes reported alterations in cytokines, cytokine receptors, acute-phase mediators, myostatin signalling, NF-κB-related mediators, and JAK/STAT pathway components identified in human skeletal muscle studies. These pathways may converge on transcriptional regulators such as FoxO and NF-κB, which are associated with the expression of catabolic mediators including MuRF1 and FBXO32/Atrogin-1, as well as regulators of protein synthesis such as mTORC1. In addition, increased expression of NLRP3 and other genes involved in inflammasome-related signalling has been reported in skeletal muscle biopsies from critically ill patients. Collectively, these findings indicate the presence of a complex inflammatory signalling network associated with altered muscle protein turnover and muscle dysfunction during critical illness. However, most available evidence derives from observational and translational studies, and therefore these associations should not be interpreted as definitive causal mechanisms. Changes in mediators, indicated with nonitalic letters, refer to protein levels, whereas those in italics denote gene expression. Arrows indicate increased or decreased gene expression, or changes in protein content, activity, or phosphorylation of inflammatory mediators and signalling proteins; arrowed lines indicate functional relationships or signalling pathways; dashed lines indicate the mediator, receptor, pathway component, or cellular process affected. * and ** indicate study-specific annotations as reported in the source studies. The symbol ? indicates an uncertain or not fully established relationship reported in the available evidence. Created in BioRender. Arellano, Ó. (2026) https://BioRender.com/4b3syhz, accessed on 2 June 2026.

Proinflammatory cytokines and acute-phase response proteins, in addition to their signalling pathways, are key mediators of inflammation-induced muscle atrophy. In conditions such as sepsis and critical illness, they primarily drive protein degradation in skeletal muscle via the UPS [77]. The main findings regarding the inflammatory response in CIM are outlined below (Figure 7).

### 7.1. Myostatin

Myostatin, a member of the transforming growth factor-β (TGF-β) superfamily, is a key regulator of skeletal muscle growth and typically functions as a negative regulator under normal physiological conditions [79]. In CIM, however, myostatin exhibits paradoxical behaviour. Increased protein levels of myostatin have been reported in VL muscle samples [70], although decreases in mRNA levels in muscle samples and plasma protein concentrations have been reported [80]. Small mother against decapentaplegic (SMAD) proteins are a family of transcription factors that function as key intracellular effectors of the TGF-β signalling pathway. Upon activation, they translocate to the nucleus to regulate the expression of genes involved in muscle atrophy [81]. In muscle samples from the TA of patients in neurointensive care units with CIM, overexpression of SMAD3 and reduced expression of SMAD6 and SMAD7 have been reported [41], indicating alterations in the myostatin receptor signalling pathway [82]. Additionally, increased nuclear localization of phosphorylated SMAD2/3 was observed in rectus femoris biopsies from patients with ICUAW [30].

### 7.2. Tumor Necrosis Factor-α (TNF-α)

TNF-α, a key cytokine involved in inflammation and homeostasis [83], is linked to skeletal muscle wasting and weakness, particularly in inflammatory conditions [77]. Elevated mRNA levels of TNF-α were documented in VL muscle biopsies from CIM patients [70]. Furthermore, upregulation of the TNF receptor family was observed in TA muscle through transcriptomic analysis [42], alongside increased protein levels of TNF receptor 1 (TNFR1) in VL muscle samples [21]. TNF-α interacts with two receptors: TNFR1, which promotes inflammation and tissue degeneration, and TNF receptor 2 (TNFR2), which facilitates local homeostasis [83]. Ligand binding to TNFR1 results in the recruitment of the adaptor molecule TNFR1-associated death domain protein (TRADD) and the assembly of several signalling complexes, including receptor-interacting serine/threonine protein kinase 1 (RIPK1), TNFR-associated factor 2 (TRAF2) and cellular inhibitor of apoptosis protein 2 (cIAP2) [83]. Consistent with this pathway, transcriptomics of TA from neuro-ICU patients with CIM revealed increases in TRADD, TRAF2, and RIPK1 [41].

### 7.3. JAK/STAT Pathway

The janus kinase (JAK)-signal transducer and activator of transcription (STAT) signalling pathway plays a pivotal role in cellular communication and involves more than 50 cytokines and growth factors, including interleukin-5,6 and 10 (IL-5, IL-6, and IL-10) [84]. In skeletal muscle, IL-6 cytokines activate this pathway, which has dual effects: it promotes muscle hypertrophy through satellite cell proliferation while also contributing to muscle wasting [85]. In this context, increases in IL-6 mRNA [70,86] and protein levels [18] were observed in CIM. Furthermore, the protein levels of IL-6 increase, along with those of other interleukins, such as IL-5, interleukin-8 (IL-8), and IL-10 [18]. In addition, increased expression of interleukin 18 receptor 1 (IL-18R1) and interleukin 10 receptor subunit alpha (IL-10RA) was observed, according to transcriptomic analysis [41]. Consequently, multiple studies have highlighted the significant role of the JAK/STAT pathway in regulating the myogenic progression of adult satellite cells [85]. JAKs interact with cytokine receptors to mediate tyrosine phosphorylation, which subsequently recruits STAT proteins. Upon phosphorylation, STAT proteins dimerize and translocate to the nucleus to regulate gene expression [84]. In muscle biopsies from the TA of critically ill patients at risk for CIM, a time-dependent increase in the protein levels of JAK1, JAK2, STAT1, and STAT3, along with the phosphorylated forms p-JAK2 (Tyr1007/1008), p-STAT3 (Tyr705), and p-JAK1 (Tyr1022/1023), was observed [87]. Additionally, increases in the protein levels of p-JAK1 (Tyr1022/1023), p-JAK2 (Tyr1007), p-JAK3 (Tyr980/981), p-STAT3 (Tyr705), and p-STAT5 (Tyr 694) were reported in the diaphragm muscle of patients undergoing mechanical ventilation [88]. JAK1 is associated with cytokines that contain the gp130 subunit, such as the interleukin-6 receptor (IL-6R). JAK2 is linked to the gp130 receptor family, which includes the interleukin-5 receptor (IL-5R), whereas JAK3 associates with all common γ chain (γc) cytokine receptors [84]. STAT1 is associated with all interferon (IFN), as well as the cytokines IL-6 and TNF, whereas STAT2 specifically correlates with IFN type I. STAT3, in turn, is linked to the IL-6 and IL-10 families of cytokines [84]. Activated STATs induce the expression of suppressor of cytokine signalling (SOCS) proteins, which bind to phosphorylated JAKs and their receptors, effectively downregulating the JAK/STAT signalling pathway [84]. Notably, increased levels of SOCS3 mRNA were documented in the VL muscle of patients at high risk of developing ICUAW [89].

In conclusion, inflammatory signalling pathways are consistently altered during critical illness and are associated with skeletal muscle dysfunction. However, interpretation of these findings requires caution, as some inflammatory mediators exhibit divergent expression patterns across studies. These differences may partly reflect variability in the muscle groups analysed, biological compartments evaluated (skeletal muscle versus circulation), timing of tissue collection, and methodological approaches used to assess gene and protein expression. Nevertheless, the recurrent activation of cytokine-related pathways, including TNF-α and JAK/STAT signalling, together with alterations in myostatin signalling, supports a potential role for inflammation in the molecular mechanisms associated with muscle wasting and weakness in critically ill patients. Further studies are needed to clarify the causal contribution of these pathways to the development of muscle dysfunction during critical illness.

## 8. The Potential Contribution of the NLRP3 Inflammasome in Muscle Dysfunction

**Figure 8:**
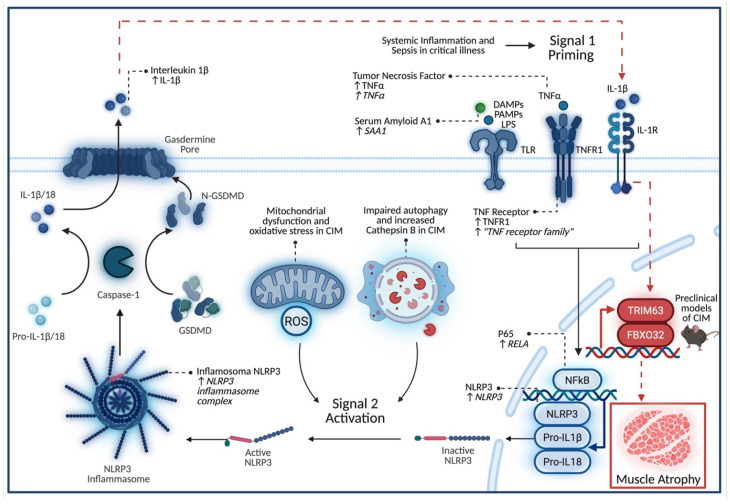
Proposed relationship between molecular alterations reported in skeletal muscle during critical illness and potential NLRP3 inflammasome signalling. Increased expression of IL-1β, TNF-α, TNF receptor family members, NF-κB-related mediators, NLRP3, and inflammasome-associated genes reported in human skeletal muscle studies may provide conditions favouring inflammasome priming (signal 1). Likewise, mitochondrial dysfunction, oxidative stress, impaired autophagy, and increased cathepsin activity observed in critically ill patients may represent biologically plausible stimuli for inflammasome assembly and activation (signal 2). Evidence from preclinical models further suggests that NLRP3 signalling may be associated with the expression of atrophy-related genes such as FBXO32/Atrogin-1 and TRIM63/MuRF1. The interactions depicted in this figure represent a proposed mechanistic framework integrating current human and experimental evidence and should not be interpreted as direct proof of NLRP3 inflammasome activation in human skeletal muscle during critical illness. Further translational studies are required to validate these relationships. Changes in mediators, indicated with nonitalic letters, refer to protein levels, whereas those in italics denote gene expression. Arrows indicate functional relationships or activation steps within the NLRP3 inflammasome pathway. The red dashed line indicates a proposed feedback loop, supported by preclinical murine models, in which cytokine secretion may reinforce signal 1 priming and additionally promote the expression of muscle atrophy-related genes. Created in BioRender. Arellano, Ó. (2026) https://BioRender.com/npgobpa, accessed on 2 June 2026.

The NOD-, LRR- and pyrin domain-containing protein 3 (NLRP3) inflammasome is a multiprotein complex essential for innate immune responses. It comprises the sensor NLRP3, the adaptor ASC (apoptosis-associated speck-like protein containing a caspase activation and recruitment domain (CARD)), and the effector caspase 1 [90,91]. Upon activation, NLRP3 oligomerizes via its NACHT domain, recruits ASC through pyrin domain (PYD) interactions, and forms ASC filaments. These filaments aggregate into a single structure (ASC speck) that recruits and activates caspase 1 through CARD–CARD interactions, enabling proteolytic processing and downstream inflammatory signalling [90]. The NLRP3 inflammasome is a key regulator of inflammatory signalling and is activated in response to pathogen-associated and damage-associated molecular patterns (PAMP and DAMP) [90]. It plays a pivotal role in the processing of IL-1β and IL-18, two crucial proinflammatory cytokines [91,92]. Although the specific involvement of the NLRP3 inflammasome in the development of CIM has not yet been elucidated, its well-established role in mediating sterile inflammation and tissue damage under other conditions suggests that it could contribute to the inflammatory and degenerative processes observed in skeletal muscle during critical illness. The main findings supporting the involvement of the NLRP3 inflammasome in the pathogenesis of CIM are described below (Figure 8).

### 8.1. Muscle Atrophy, Inflammation, and the NLRP3 Inflammasome

Muscle atrophy and weakness, hallmark features of CIM, are strongly associated with systemic inflammatory states such as sepsis and SIRS, as well as elevated levels of inflammatory mediators [93]. The key proinflammatory cytokines implicated in muscle degradation include TNF-α, IL-6, and IL-1 [77]. In addition to systemic inflammation, skeletal muscle itself functions as a secretory organ capable of producing and releasing various myokines—such as IL-6 and IL-1β—which may further modulate local and systemic immune responses and contribute to muscle wasting [77]. The cleavage and secretion of IL-1β are regulated by the activation of the NLRP3 inflammasome [92]. In addition, evidence highlights the role of the NLRP3 inflammasome not only as a central regulator of muscle metabolism [94,95,96] but also in the pathogenesis of various myopathies [95,97,98], and sepsis-induced muscle atrophy in C2C12 myotubes [99]. In relation to CIM, in preclinical models of this condition, the NLRP3 inflammasome was implicated in muscle atrophy, as it regulates the upregulation of ubiquitin ligases such as MuRF1 and atrogin-1, which are associated with muscle protein degradation [100,101]. The upregulation of these ubiquitin ligases has been extensively documented in human CIM [4,16,18,29,42,46,70,78]. Given that experimental models reproduce several structural and molecular features observed in human CIM, these findings support the hypothesis that NLRP3 inflammasome activation may contribute to muscle atrophy during critical illness. However, direct evidence of NLRP3 activation in human skeletal muscle from patients with CIM remains limited.

### 8.2. Factors Potentially Promoting NLRP3-Mediated Muscle Dysfunction in Critical Illness

Activation of the NLRP3 inflammasome occurs in two steps. The first step, termed “priming,” is initiated through the recognition of PAMP or DAMP by pattern recognition receptor (PRR) [90]. This process is also initiated by the stimulation of cytokine receptors such as TNFR and interleukin-1 receptors [90]. Notably, increased protein levels and overexpression of IL-1β, TNF-α, and TNFR have been observed in the muscle of patients with CIM and critical illness [18,21,42,70,78], which may indicate inflammasome activation. Transduction of these receptors triggers the activation of nuclear factor kappa-B (NF-κB), which subsequently enhances the transcription of NLRP3, pro-IL-1β, and pro-IL-18 [90,91,102]. NF-κB is a family of inducible transcription factors that regulate a broad array of genes involved in the inflammatory response [103]. The NF-κB family includes five structurally related members: NF-κB1 (p50), NF-κB2 (p52), RelA (p65), RelB, and c-Rel [103]. The I-kappaB kinase (IKK) complex, which is composed of IKKα, IKKβ (the kinases), and NF-κB essential modulator (NEMO), IKKγ, the regulatory subunit, plays an essential role in the NF-κB signalling cascade. TGF-beta-activated kinase 1 (TAK1) phosphorylates IKKβ, a crucial step in canonical NF-κB signalling [103,104]. In TA muscle samples from neuro-ICU patients, transcriptomic analysis revealed an upregulation of components of NF-κB signalling, including RelA and NEMO, alongside a reduction in TAK1-binding protein 2 (TAB2) [41]. This pattern suggests that NF-κB activation is involved in the inflammatory processes associated with CIM. The NLRP3 inflammasome can be activated by various inflammatory and pathogen-associated signals [91,105]. Given that sepsis and SIRS are common risk factors for CIM [2,9,10,39,106,107,108], critically ill patients provide a favourable environment for inflammasome priming. Increased expression of TNF-α, TNFR, and IL-1β was observed in the muscles of patients with CIM [109]. Furthermore, the overexpression of genes associated with the NF-κB pathway has been shown in the TA muscle of neuro-ICU patients [41] and murine ICU models [110], suggesting that NF-κB plays a critical role in CIM development. Collectively, these findings suggest that conditions favouring NLRP3 inflammasome priming may be present during critical illness and CIM. However, direct demonstration of inflammasome priming and activation in human skeletal muscle remains to be established. The second step involves the assembly of the NLRP3 complex, culminating in the activation of caspase-1 [90,91,102]. This enzyme cleaves pro-IL-1β and pro-IL-18 into their active forms, leading to an inflammatory cascade, including pyroptotic cell death [90,91,102,111]. Mitochondrial dysfunction and the generation of ROS are stimuli for NLRP3 inflammasome activation [90,91,102]. Given the well-documented presence of mitochondrial dysfunction and oxidative stress in CIM [4,20,25,29,49], this represents a biologically plausible context for NLRP3 inflammasome activation. Nevertheless, direct evidence demonstrating activation of this complex in human skeletal muscle during CIM is still lacking. Potassium efflux, which is mediated by NIMA-related kinase 7 (NEK7), also triggers the second activation of the inflammasome complex [90,91,102]. In human macrophages, serum amyloid A1 (SAA1) has been shown to activate the purinergic receptor P2 × 7, resulting in increased potassium efflux [77]. SAA1, a key component of the acute-phase response activated by stressors such as tissue injury, infection, and surgery, plays a crucial role in lipid metabolism and inflammation regulation [112]. Additionally, SAA1 activates the toll-like receptor (TLR) 2/4-NF-κBp65 signalling pathway, contributing to myocyte atrophy [113]. Increased SAA1 mRNA levels were observed in VL muscle samples from patients at high risk of developing ICUAW [86]. Furthermore, the release of lysosomal contents, such as cathepsin B, contributes to the second activation of the NLRP3 inflammasome [91,114]. As mentioned previously, increased levels of cathepsins and their activity were reported in CIM [4,42,46]. Thus, it is logical to connect mitochondrial dysfunction, elevated oxidative stress, and the involvement of cathepsins in CIM with the second activation of the NLRP3 inflammasome. However, this relationship has yet to be thoroughly investigated in CIM. The NLRP3 inflammasome represents a biologically plausible pathway that may contribute to muscle dysfunction during critical illness; however, direct evidence in human CIM remains limited. In addition to the upregulation of key cytokines, inflammatory pathways, such as those mediated by the JAK/STAT and NF-κB signalling pathways, contribute significantly to the muscle wasting and dysfunction observed in critically ill patients. Given its role in regulating muscle protein degradation, the NLRP3 inflammasome may further link inflammation to muscle atrophy in CIM. Understanding the potential involvement of the NLRP3 inflammasome in CIM development could provide insights into new therapeutic strategies to prevent or mitigate muscle loss in critically ill patients.

## 9. Conclusions and Perspective

ICUAW is a prevalent complication in intensive care units, contributing to the deterioration of respiratory and motor functions, increasing hospital costs, and resulting in long-term cognitive, mental, and physical impairments that negatively affect the quality of life of surviving patients. Despite ongoing research into the molecular mechanisms underlying muscle dysfunction during critical illness and efforts to develop preventive interventions, the full understanding of the molecular pathways involved remains incomplete.

Overall, several molecular alterations consistently observed in critically ill patients, including activation of inflammatory pathways, mitochondrial dysfunction, oxidative stress, lysosomal perturbations, and increased expression of cytokines associated with NLRP3 priming, support the biological plausibility of inflammasome involvement in muscle dysfunction during critical illness. However, most evidence linking NLRP3 to muscle wasting derives from experimental models, other muscle disorders, or indirect mechanistic observations. Therefore, while the NLRP3 inflammasome represents a promising candidate pathway linking inflammation to muscle dysfunction, its specific contribution to human critical illness myopathy remains hypothetical and requires direct experimental validation.

## 10. Knowledge Gaps and Future Research Priorities

Despite significant advances in understanding the molecular mechanisms underlying muscle dysfunction during critical illness, important knowledge gaps remain. Evidence supporting mitochondrial dysfunction, activation of the ubiquitin–proteasome system, autophagy-related alterations, and inflammatory signalling is largely derived from human skeletal muscle studies. In contrast, the proposed involvement of the NLRP3 inflammasome remains largely hypothetical, as direct evidence demonstrating its activation in human skeletal muscle during critical illness is currently lacking. Future studies should focus on directly evaluating NLRP3 inflammasome activation in skeletal muscle obtained from critically ill patients and determining its relationship with mitochondrial dysfunction, proteolytic pathways, autophagy, and functional recovery. Importantly, recent translational research initiatives have begun to address these knowledge gaps through the integration of skeletal muscle biopsies, molecular analyses, and clinical outcomes in critically ill patients [115].

## Data Availability

No new data were created or analyzed in this study. Data sharing is not applicable to this article.

## Abbreviations

The following abbreviations are used in this manuscript:

4E-BP	eIF4E-binding protein.
AMBRA1	activating molecule in beclin-1-regulated autophagy.
ASC	apoptosis-associated speck-like protein containing a CARD
ACTN3	actinin 3.
AMPK	adenosine monophosphate-activated protein kinase.
ATG5/7/8/12	autophagy-related proteins 5/7/8/12, respectively.
ATP	adenosine triphosphate.
ICUAW	intensive care unit-acquired weakness.
Bcl-2	B-cell CLL/Lymphoma 2.
Bnip3	Bcl-2 E1B protein-interacting protein 3.
CARD	caspase activation and recruitment domain.
CAT	catalase.
CI/II/III/IV/V	complexes I/II/III/IV/V of the electron transport chain, respectively.
CIM	critical illness myopathy.
CIP	critical illness polyneuropathy.
CMA	chaperone-mediated autophagy
CTS A/B/D/S/Z	genes encoding cathepsins A/B/D/S/Z, respectively.
DAMP	damage-associated molecular pattern.
DNA	deoxyribonucleic acid.
DRP1	dynamin Related Protein 1.
eIF2B	eukaryotic initiation factor 2B.
eIF4E	eukaryotic initiation factor 4E.
eIF4G	eukaryotic initiation factor 4G.
FIS1	mitochondrial fission 1 protein.
FoxO	forkhead Box O.
GABARAPL1	GABA type A receptor-associated protein like 1.
GPx	glutathione peroxidase.
GSK3	glycogen synthase kinase 3.
IFN	interferon.
IGF-1	Insulin-like growth factor 1.
IKK	I-Kappaβ Kinase.
IL-18	interleukin-5/6/8/10/18, respectively.
IL-1β	interleukin-1 beta.
IL-5R	interleukin-5 receptor.
IL-6R	interleukin-6 receptor.
IL-10RA	interleukin-10 receptor subunit alpha.
IL-18R1	interleukin-18 receptor 1.
JAK	janus kinase.
IMM	inner mitochondrial membrane.
IRS-1	insulin receptor substrate 1
LAMP-2A	lysosomal-associated membrane protein 2A.
LC3	microtubule-associated protein light chain 3.
MAP1LC3B	microtubule-associated protein light chain 3B gene.
MFF	mitochondrial fission factor.
MFN1/2	mitofusin 1/2, respectively.
MID49/51	mitochondrial dynamics of 49 and 51 kDa.
MOF	multiple organ failure.
mRNA	messenger RNA.
mtDNA	mitochondrial Deoxyribonucleic Acid.
mTOR	mammalian target of rapamycin.
mtSODs	mitochondrial superoxide dismutases.
MuRF1	muscle RING finger 1.
MUSA1:	muscle ubiquitin ligase of the SCF complex in atrophy—1.
MyBP-C	myosin-binding protein C.
MYOM	myomesin.
MYOT	myotilin.
NAF-1	nutrient-deprivation autophagy factor 1.
NEK7	NIMA-related kinase 7.
NEMO	NF-κB essential modulator.
NF-κB	nuclear factor kappa B.
NLRP3	NOD-,LRR- and pyrin domain-containing protein 3.
NRF1/2	nuclear respiratory factor 1/2, respectively.
OMM	outer mitochondrial membrane.
OPA1	optic atrophy protein 1.
OXPHOS	oxidative phosphorylation.
PAMP	pathogen-associated molecular patterns.
PDK1	3′-Phosphoinositide-dependent kinase 1.
PE	phosphatidylethanolamine.
PGC-1α	peroxisome proliferator-activated receptor gamma coactivator 1-alpha.
PGC-1β	peroxisome proliferator-activated receptor gamma coactivator 1-beta.
PI3K	phosphatidylinositol 3-kinase.
PI3KCIII	class III phosphatidylinositol 3-kinase complex.
PICS	post intensive care syndrome.
PINK1	PTEN induced kinase 1.
PIP_3_	phosphoinositide 3,4,5-triphosphate.
PPARγ2	peroxisome Proliferator-Activated Receptor gamma-2
PRDX3	peroxiredoxin 3.
PSMB 1/8	proteasome Subunit Beta Types 1/8, respectively.
PYD	pyrin domain.
RIPK1	receptor interacting serine/threonine protein kinase 1.
RNA	ribonucleic acid.
REDD1	regulated in development and DNA damage response 1.
ROS	reactive oxygen species.
S6K1	p70S6 kinase 1.
SAA1	serum amyloid A1.
SDH	succinate dehydrogenase.
SIRS	systemic inflammatory response syndrome.
SMAD	small mother against decapentaplegic.
SOD	superoxide dismutase.
SOCS	suppressor of cytokine signalling.
STAT	signal transducer and activator of transcription.
TA	tibialis anterior
TAB 2	TAK1-Binding Proteins 2.
TFAM	mitochondrial transcription factor A.
TGF-β	transforming growth factor-β
TLR:	toll-like receptor.
TNFR1/2:	TNF-α Receptor 1/2, respectively.
TRADD	TNFR1-associated death domain protein.
TRAF2	TNFR- Associated Factor 2.
TRIM62	tripartite motif-containing 62.
TSC2	tuberous sclerosis complex 2.
ULK1	unc-51-like kinase 1.
UPS	ubiquitin proteasome system.
USP10	ubiquitin Specific Peptidase 10.
UVRAG	UV radiation resistance-associated.
VIDD	ventilator-induced diaphragmatic dysfunction.
VL	vastus lateralis
VPS34	vacuolar protein sorting 34.
